# Vale Atque Salve

**Published:** 1991-12

**Authors:** 


### Vale Atque Salve

The editor wishes to thank all those who have contributed to the Bristol Medico-Chirurgical Journal and to its successor, The West of England Medical Journal, during the past ten years of his editorship. The function of editor is merely that of a midwife in bringing into the world the fruits of the labour and enterprise of others, but for all that it has its pleasures and rewards. He wished particularly to record his satisfaction that Dr. Paul Goddard, assistant editor since 1985, whose energy and dynamism have contributed so much, has taken over the editorship and that Professor John Farndon, who provided the essential leadership in our transformation will continue as Chairman of the Editorial Board.

